# Effects of Granulated Cork with Bark on the Microstructure and Resistance to Extreme Environmental Conditions of Concrete for Non-Structural Precast Elements

**DOI:** 10.3390/ma18050933

**Published:** 2025-02-21

**Authors:** María Concepción Pacheco Menor, María José Arévalo Caballero, Antonio Macías García, Pedro Serna Ros

**Affiliations:** 1 INTROMAC—Technological Institute of Ornamental Rocks and Building Materials, Campus Universidad de Extremadura, 10071 Cáceres, Spain; 2 Polytechnic School, University of Extremadura, Avda. de la Universidad, s/n, 10004 Cáceres, Spain; 3 School of Industrial Engineerings, University of Extremadura, Avda. de Elvas, s/n, 06071 Badajoz, Spain; 4 ICITECH—Institute of Concrete Science and Technology, Universitat Politècnica de València, 4N Building, Camino de Vera s/n, 46022 València, Spain

**Keywords:** bio-aggregates, granulated cork with bark, non-structural precast elements

## Abstract

The building sector is responsible for major environmental impacts. Utilising bio-based raw materials, such as bio-aggregates, in concrete production could address to this environmental challenge. While the physical and mechanical properties of various bio-based concretes have been explored, research on their microstructure and resistance to extreme conditions is limited. Cork is a light, renewable and biodegradable material. Cork industries produce a considerable number of solid wastes, among them is granulated cork with bark (GCB) that is not adequate to produce agglomerated cork. To reduce this waste volume, it is possible to use GCB as a bio-based aggregate in the production of concrete for applications in non-structural precast elements that are lighter and/or have thermal properties. The influence of GCB on the microstructure and resistance to extreme conditions of concrete for non-structural use is presented here. Concrete mixes with GCB are compared with a concrete mix made with natural aggregates (RC). Replacements of 20% and 30% of natural aggregate (2–5 mm) by GCB were considered. The microstructure shows the good integration of the GCB in the cement matrix. Freeze–thaw and wet–dry cycle tests do not affect the variation in mass and compressive strength of concrete mixes with GCB in comparison to RC mixes, although they do affect its visual appearance and microstructure somewhat. Concrete mixes with GCB present a greater variation in mass and compressive strength, 30% for RC mix and 43–49% for concrete mixes with GCB, under high temperatures. Concrete mixes with GCB did not show spontaneous combustion.

## 1. Introduction

The building sector significantly impacts on the environment, primarily through the consumption of non-renewable raw materials, greenhouse gas emissions and waste production. Consequently, the development of new sustainable building materials has become a global priority to contribute to the reduction in climate impact of construction [1]. Concrete is the most extensive and generally used construction material worldwide [2]. However, the manufacture of this material represents an unsustainable production model due to the use of cement and aggregates in its composition. Industrial wastes, such as fly ash [3], ground granulated blast furnace slag [4], waste paper, pulp waste paper or paper sludge ash [5,6] have been explored to replace cement. On the other hand, the use of construction and demolition waste as aggregates in concrete is well-documented. Studies on other by-products or wastes and their effect on detrimental processes have also been evaluated. For instance, He et al. [7] studied the freeze–thaw resistance of concrete incorporating waste rubber or waste glass as aggregates. Rubberized concrete, as opposed to glass concrete, outperformed reference concrete in reducing apparent damage and mass loss during freeze–thaw cycles. Furthermore, rubberized concrete demonstrated superior freeze–thaw resistance, mechanical strength, and a denser microstructure compared to reference concrete. González-Ortega et al. [8] found that after wet–dry cycles, water mobilised products from corroded iron nodules present in the electric arc furnace slag, depositing them on concrete surfaces and increasing stained points, while reference specimens remained unaltered. EPS waste has also been considered as an aggregate for concrete. Even after burning, bonding is still possible with the composing material [9].

In recent years, bio-based materials have gained attention as aggregates in concrete manufacturing due to their renewable nature and their generation as sub-products of local agricultural activities and related industries [10]. Compared to conventional concrete, bio-based concrete generally has lightweight characteristics and is associated with better thermal and sound absorption properties, attributed to the micropores in bio-aggregates [11]. Diverse bio-based aggregates such as apricot shells [12], peach shells [13], oil palm shells [14,15], coconut shells [16], corn stalks [17], hemp [18], bamboo [19], wood [20], cork [21], etc., have being successfully used as aggregates in concrete production.

Current research on bio-based concrete mainly examines its physical properties and mechanical properties. However, there are limited studies on how bio-aggregates affect microstructure and durability under extreme conditions, such as fire exposure or freeze–thaw resistance [22], particularly in concrete made with granulated cork. SEM micrographs of the ITZ of concrete made with apricot and peach shells have shown cracks between the shell and the mortar interface attributed to the organic nature and smooth surface of the shells [23,24]. Similar issues have been observed in wood sand concrete [25] and oil palm shell concrete [26].

In terms of resistance towards elevated temperature, concrete generally loses significant strength when exposed to temperatures above 400 °C. For concrete made with an oil palm shell aggregate, the compressive strength loss at 200 °C and 400 °C has been reported to be around 30–35% and 55–75%, respectively [27,28]. Similarly, coconut shell coarse aggregate concrete shows a strength loss of about 25–50% at 200 °C and 60–80% at 400 °C, depending on the exposure duration [29]. These concretes also exhibit colour changes when subjected to high temperatures. Oil palm shell and coconut shell concrete turn light brown after 1 h at 400 °C, and light yellow and light grey after 2 and 3 h, respectively [28,29]. Jumaat et al. [30] observed that their concrete surface turned slightly reddish after 1 h at 500 °C due to the partial burning of the oil palm shell aggregate.

The effect of immersion weathering on corn stalk concrete made with magnesium phosphate cement has been studied by Ahmad et al. [22]. They found that mass loss increased with the number of immersion cycles, attributed to microstructure weakening and increased porosity. Additionally, a decrease in sample area and compressive strength was notable after the immersion weathering. The authors also investigated the frost resistance of corn stalk concrete. Despite no visible signs of surface cracking, mass loss and reduced compressive strength were observed, primarily due to binder leaching during the freeze–thaw test.

Regarding the effect of long-term exposure to dry and humid environments on the durability of lightweight wood chipping concrete, a strength loss was observed in samples stored in both ambient and humid atmospheric conditions. This deterioration is particularly pronounced under humid conditions [31]. Despite this, woodchip-based concrete maintains reasonable strength and durability after 16 months of exposure regardless of the environment, meeting the class III RILEM specification for lightweight concrete.

Cork is a light, renewable and biodegradable material with a homogeneous cellular structure formed by regularly arranged cell walls without intercellular space. This structure, along with its chemical composition containing the lipophilic macromolecule suberin, makes cork a bio-based material with unique properties. It has a low density, viscoelastic behaviour, low thermal conductivity, and good sound absorption [32]. Cork oak forests are species-rich ecosystems, and cork extraction provides a valuable product while preserving the biological system. Cork industries produce diverse by-products: granulated cork, expanded cork and granulated cork with bark (GCB). Although GCB is unsuitable for producing cork agglomerates, it has properties that make it suitable as a bio-aggregate in concrete [33]. The physical and mechanical properties of concrete with different types of by-products of cork have been analysed. The authors [34] found that concrete mixtures with GCB have lower absorption by capillary and density compared with a conventional reference concrete. Despite a reduced mechanical strength, this material remains useful for non-structural purposes. Notably, mixes with GCB have a lower maximum thermal conductivity than reference concrete. Regarding the long-term durability of cork concrete, only one study has examined this property, and it focused on one by-product and one property, the resistance to freeze–thaw. Branco et al. [35] conducted a comparative study on the performance of three types of concrete: standard concrete, concrete with an air-entraining admixture and cork concrete. Their findings indicated that incorporating 4% granulated cork by volume into concrete does not provide an effective alternative to the air voids generated by an air-entraining agent under freeze–thaw cycles. However, granulated cork may enhance the performance of concrete when exposed to temperature fluctuations.

**Figure 1:**
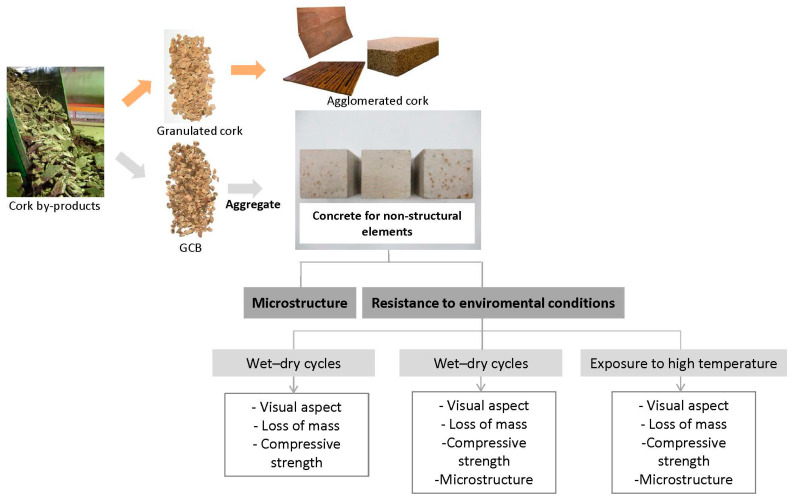
Methodology of the study.

Evaluating the impact of detrimental processes on building materials is particularly important, especially for bio-based concrete. Therefore, given the limited research on the long-term durability of concretes made with bio-aggregates, particularly cork-derived ones, there is a need for further studies to better assess the performance of bio-based concretes. This paper aims to investigate the microstructure and resistance to extreme conditions of concrete produced with GCB for future applications in non-structural precast pieces such as solid or hollow blocks that are lighter and have better thermal properties with decreased volumes. The influence of the replacement level of natural aggregates by GCB in concrete mixes has been evaluated regarding mass loss and compressive strength after freeze–thaw cycles or exposure to high temperatures and concrete performance after wet–dry cycles. The evolution of visual features and the microstructure of concrete are also examined in this study (Figure 1).

## 2. Materials and Methods

### 2.1. Materials

**Figure 2:**
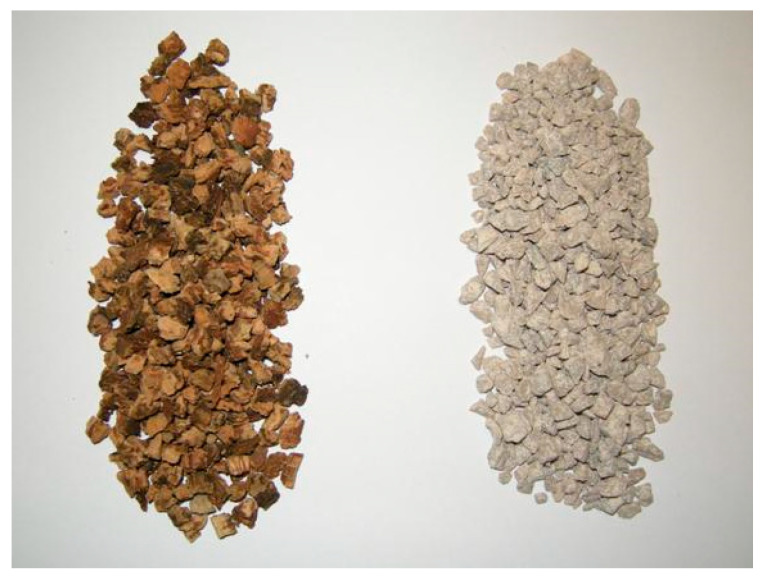
GCB 3.2–5 mm (**left**) and limestone raw aggregate 2–5 mm (**right**).

**Figure 3:**
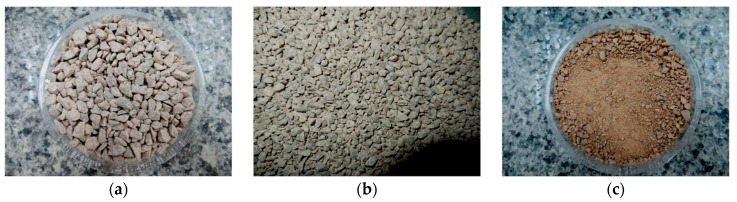
Limestone raw aggregates: (**a**); 4–8 mm; (**b**) 2–5 mm; (**c**) 0–4 mm.

Portland-limestone cement (CEM II/A-L 42.5 R) (Cementos Balboa, Spain) as used as a binder. The characteristics of this cement complied with European EN 197-1:2011 [36]. GCB produced and classified by Eurocork Almendral S.L. (Almendral, Spain) of size 3.2–5 mm and high density (Figure 2), and three fractions of limestone raw aggregates (0–4, 2–5 and 4–8 mm) (Figure 3) were used as aggregates for concrete production.

The properties of the GCB were obtained in a previous study [33]. Granulometric analyses of the GCB and limestone raw aggregates were performed as stated by ISO 2030:2018 [37] and EN 933-1:2012 [38] (Table 1). The physical properties were estimated, conforming to EN-1097-6:2022 [39] (Table 2).

In the previous study carried out by the authors of this article, GCB showed an appropriate shape and texture to be utilised as an aggregate in concrete. There were two prevalent shapes, flaky (22%) and irregular (78%). GCB texture might be considered rough in 100% of grains [33].

### 2.2. Mix Proportions

**Figure 4:**
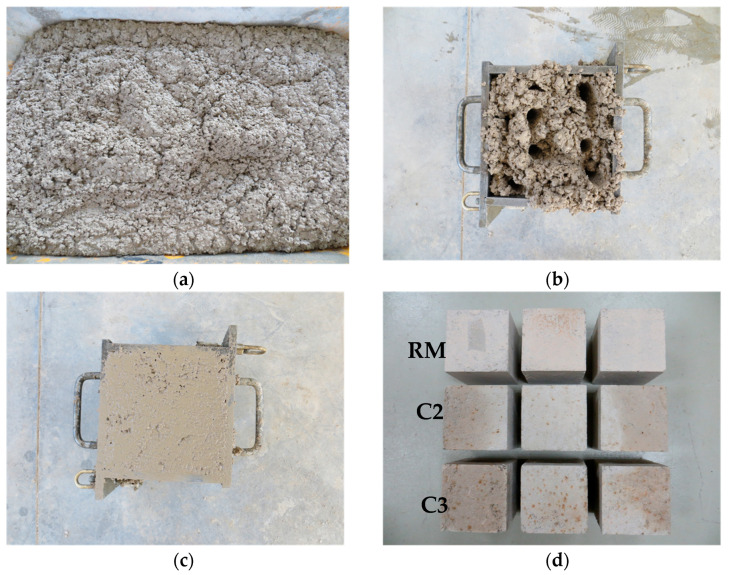
Concrete mix preparation: (**a**) C3; (**b**) specimen of compacted C3; (**c**) specimen of compacted C3 after being hit with a hammer and roughed; (**d**) unmoulded concrete mix specimens.

A cement content of 182 kg/m^3^, with industrial dosage utilised in the production the of concrete for the non-structural blocks, was considered to design the concrete mixtures. Three concrete mixes were prepared: one without GCB (RC) and two mixes with different GCB proportions, replacing 20% (C2) and 30% (C3) of 2–5 mm aggregate with an equal volume of GCB (Figure 4). The total water-to-cement (*w*/*b*) ratio and the substitution rates were determined according to a previous study [35]. A *w*/*b* ratio of 1.2 was the lowest needed for suitable workability under laboratory conditions. Concrete mix (RC) was manufactured with dosage substitution rates of 20% and 30% provided acceptable performance. Although the C2 and C3 mixes exhibited reduced compressive strength with respect to the RC mix, the compressive strength values were still suitable for non-structural elements. In addition, C2 and C3 showed improvements in properties such as thermal conductivity and capillary water absorption. Detailed concrete mix proportions are shown in Table 3. All samples were made from the same GCB batch, which was previously dried at 60 °C to remove moisture. The bulk density was also evaluated (209.9–229.3 kg/m^3^) [29]. Before manufacturing each batch of concrete, the t bulk density of the GCB sample was checked again. As the GCB was already classified at the factory based on its density, the sample of this material was homogeneous.

### 2.3. Casting and Curing Conditions

The fresh concrete was deposited in moulds and it was compacted by hand to cubic specimens of 100 mm size in one layer and to cubic specimens of 150 mm size in two layers. Then, the specimens were introduced into a humid chamber at a temperature of 20 °C and a relative humidity of 95% until they reached the testing age, according to EN 12390-2:2019 [40].

### 2.4. Test Procedures

#### 2.4.1. Microstructure

The microstructure morphology of various samples was studied by means of scanning electron microscopy (SEM). This kind of analysis enabled us to visualise matrix–aggregate adhesion within various composites [23]. To facilitate observation, the samples were initially dried and then covered with a thin layer of spray-on platinum, which acted like a conductor. The micrographs were performed using a Hitachi S-4800 microscope (Hitachi High-Technology Corporation, Japan). An energy dispersive X-ray analyser (EDAX) was used together with SEM. EDAX analysis allowed us to make an elementary analysis of chemical species (qualitative study).

#### 2.4.2. Frost Resistance

**Figure 5:**
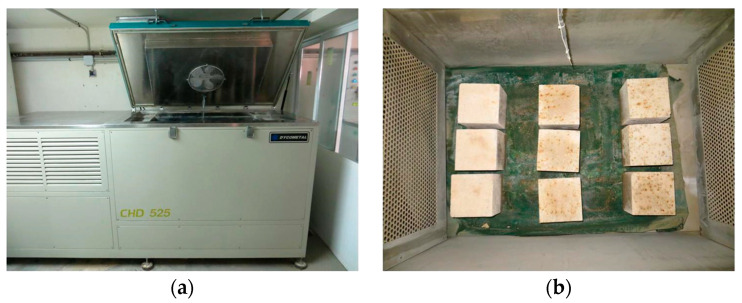
Frost resistance test: (**a**) climatic chamber; (**b**) specimens in air-freezing period.

The determination of frost resistance was performed by an adaptation of the procedure taken from EN 12371:2010 [41]. After curing for 28 days, six cube specimens (150 × 150 × 150 mm^3^) were chosen: three specimens were used to assess compressive strength according to EN 12390-3:2019 [42] and the other three specimens were weighted and subjected to 48 freeze–thaw cycles, performed in a Dycometal CHD-525 (Dycometal Testing Technology, Barcelona, Spain) (Figure 5) according to EN 12371:2010 [41].

After 48 cycles, the specimens were air-dried at >15 °C and RH < 60% in a controlled environment chamber to constant mass and were visually inspected to detect cracking, surface scaling or colour change. After that, the specimens were kept in a chamber at 20 °C and 95% up to constant mass and the mean values of mass and compressive strength were used to evaluate the frost resistance of the concrete.

#### 2.4.3. Resistance to Ageing by Thermal Shock

To investigate the resistance of concrete with CGB to thermal shock, the EN 14066:2013 [43] method was followed. This standard proposes an analysis of this property through wet–dry cycles to simulate the behaviour of to rain filtration or the change in the water table in the real environment. This procedure also allowed us to analyse the effect of leaching products on the GCB during the wet–dry processes. After curing for 28 days, six cube specimens (150 × 150 × 150 mm^3^) were chosen: three specimens were used to assess compressive strength according to EN 12390-3:2019 [42] and the other three specimens were weighed and subjected to 20 wet–dry cycles.

In a wet–dry cycle, specimens were fully immersed in water for 6 h and then dried for 18 h at 105 °C. At the end of the cycles, a visual inspection was performed to identify any type of wear or colour change on the surface. Then, specimens were kept in a chamber at 20 °C and 95% up to constant mass and the mean values of mass and compressive strength were used to evaluate the resistance of the concrete to ageing by thermal shock. The mass was evaluated before and after 20 wet–dry cycles. The microstructure of the specimens’ surface was analysed by SEM as described in Section 2.4.1. Three randomly chosen areas on the samples were selected for analysis.

#### 2.4.4. Exposure to High Temperatures

**Figure 6:**
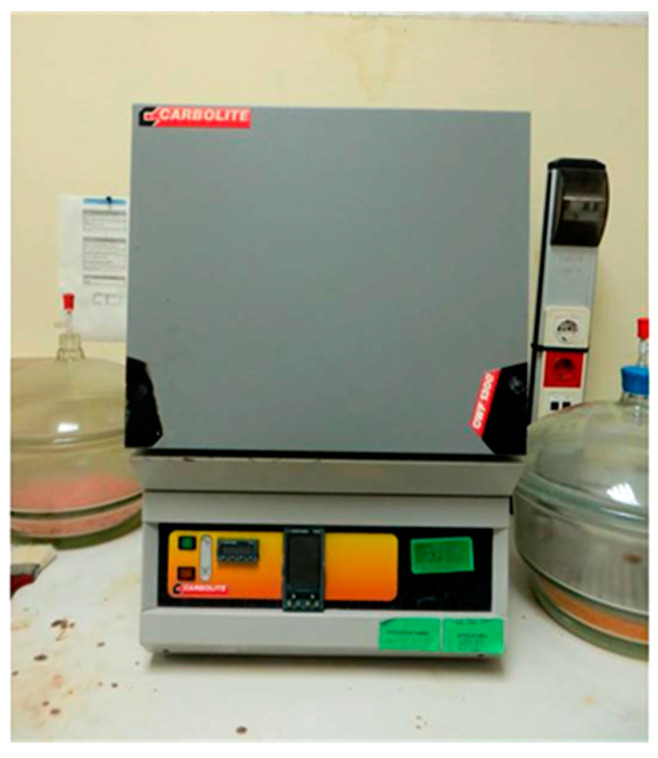
Electric furnace.

The procedure adopted here was based on that defined by He and Song [44]. Three cubic specimens (100 × 100 × 100 mm^3^), cured for 28 days and dried at 80 °C to constant mass, were used to evaluate the durability of concrete exposed to high temperatures by means of their visual aspect, mass, compressive strength and microstructure evaluation. Specimens were exposed to 600 °C in an Carbolite CWF 1300 (Hope, Derbyshire, United Kingdom) for 6 h and heating was performed at a rate of 16 °C per min (Figure 6).

The cooling process began by keeping the specimens in the turned-off electric furnace for 1 h. Then, the specimens were cooled naturally to room temperature. Mass differences in specimens were evaluated before and after exposure to high temperatures and the compressive strength was compared to three specimens dried at 80 °C. The microstructures of the specimens’ surfaces were analysed by SEM as described in Section 2.4.1. Three randomly chosen areas on the samples were selected for analysis.

## 3. Results and Discussion

The results of the porosity and mechanical properties from a comprehensive analysis of each concrete mix, conducted in a previous study, are summarised in Table 4 and Table 5 [34]. As generally happens when lightweight aggregates with high water absorption are used [45], there is an increase in the total porosity with respect to the RC mix and 0.1–100 µm pores are larger in C2 and C3. The addition of GCB gives rises to a decrease in flexural and compressive strength in concrete mixes, affected by the concrete porosity, the strength of the aggregate, the strength of the cement paste [46] and the adhesion between the cement paste and the aggregate surface [47].

### 3.1. Effects of GCB on Microstructure of Non-Structural Concrete

**Figure 7:**
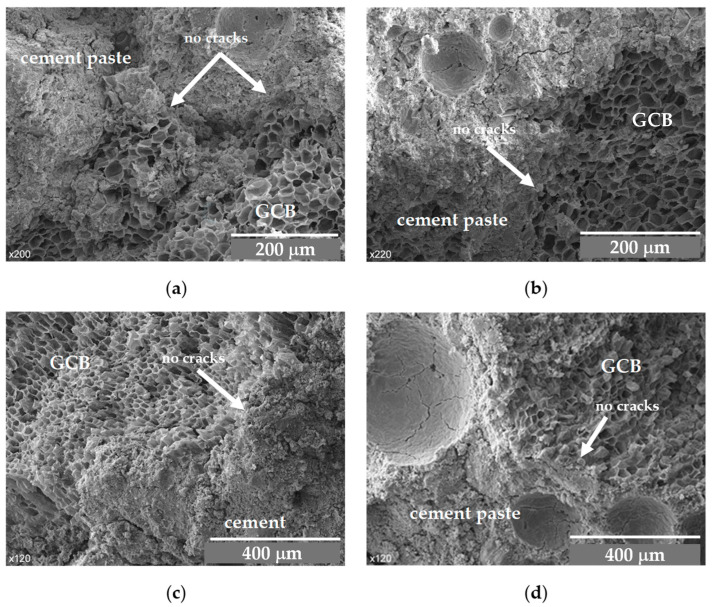
SEM images of GCB and cement paste at 7 days: (**a**) C2 × 200 magnification; (**b**) C2 × 220 magnification; (**c**) C3 × 120 magnification; (**d**) C3 × 120 magnification.

Generally, there is a weak bond between organic aggregates and mortar [23], probably due to the different nature of the two materials. However, Figure 7 shows a continuous structure in which the GCB appears surrounded by cement matrix at an age of 7 days. There is, however, a defined contact zone that separates the two materials, cement paste and GCB. The organic material retains its cellular structure, although the cells of the GCB appear a little deformed, possibly due to the pressure exerted by the cement paste on the aggregates.

**Figure 8:**
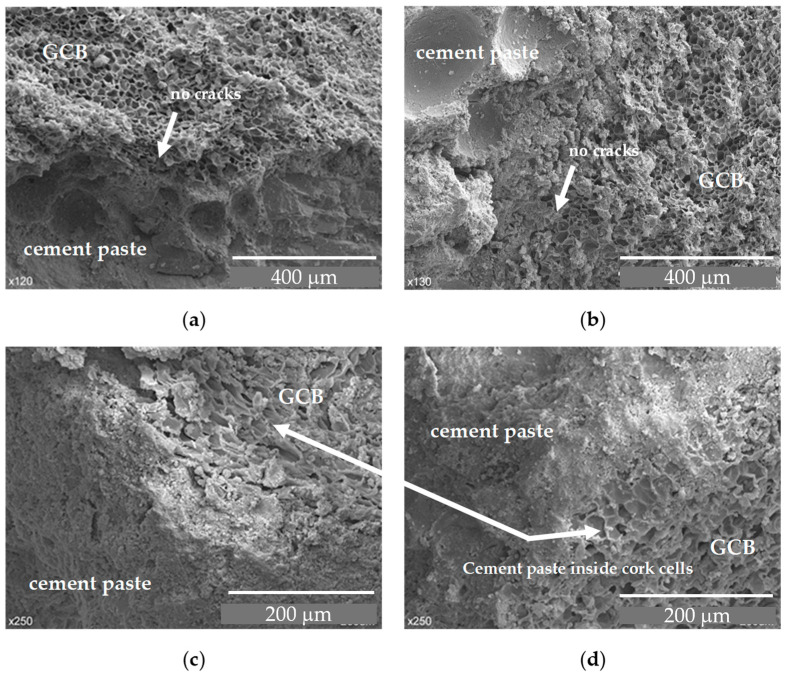
SEM images of GCB and cement paste at 28 days: (**a**) C2 × 120 magnification; (**b**) C2 × 130 magnification; (**c**) C3 × 250 magnification; (**d**) C3 × 250 magnification.

At 28 days of age, the concrete structure maintains a similar appearance to that at 7 days age and the GCB granules retain their cellular structure. The cement paste seems to be well adhered to the surface of the GCB and some cement paste can be even seen inside cells (Figure 8) which can improve the microstructure bonding properties.

**Figure 9:**
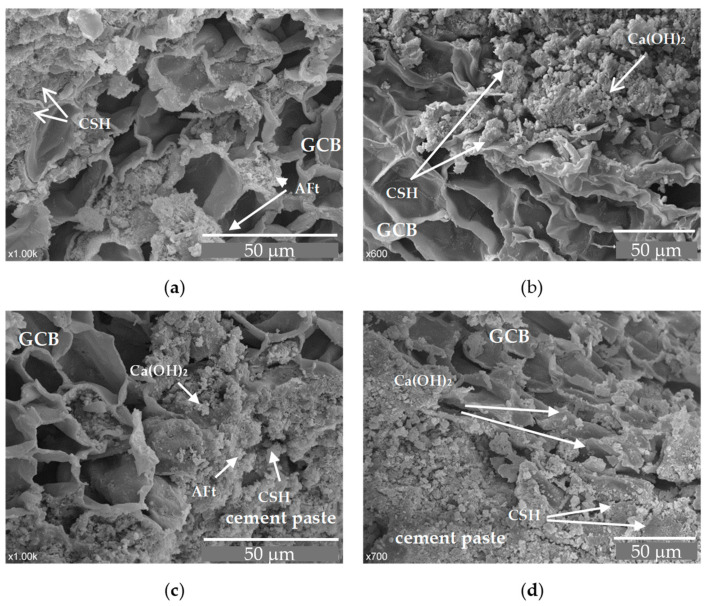
SEM images of GCB and cement paste: (**a**) C2 × 1000 magnification, at 7 days; (**b**) C3 × 600 magnification, at 7 days; (**c**) C2 × 1000 magnification, at 28 days; (**d**) C3 × 700 magnification, at 28 days.

In the concrete with the GCB, two materials of different chemical properties come into contact, cement and the GCB surface, giving rise to a contact area with a complex microstructure. The morphology of this area in the concrete mix samples with GCB at 7 days old is shown in Figure 9a,b, and at 28 days old in Figure 9c,d.

These images show some structures of amorphous appearance in the cement paste next to the GCB granules and inside the cells, possibly due to deposits of calcium silicate hydrate (CSH). The structures in Figure 3a,b exhibit a higher porosity than those in Figure 9c,d, where the structure seems more compact and denser, owing to the development of CSH gel. Figure 9a, corresponding to the C2 mix, shows particles with an acicular form, presumably due to calcium aluminate ferrite trisubstituted (AFt) phases. Smaller quantities of these structures are observed in Figure 9c, probably due to a transformation from the AFt phases to aluminate ferrite monosubstituted (AFm) phases because of the evolution of the cement hydration process [48]. The cement hydration seems to have experienced some delay, after seven days of curing there were still many acicular structures. This fact, that in a normal curing process occurs after 1–3 days, in this material might be explained by the ability of the GCB’s walls to absorb water in the mixing process and then to gradually release the water towards the cement paste, thus keeping the cement components dissolved for a longer time, among them being the sulphate ions that favour the formation of calcium AFt phases [49].

In the SEM image corresponding to C3, acicular structures are not observed, probably due to the greater ratio of GCB.

**Figure 10:**
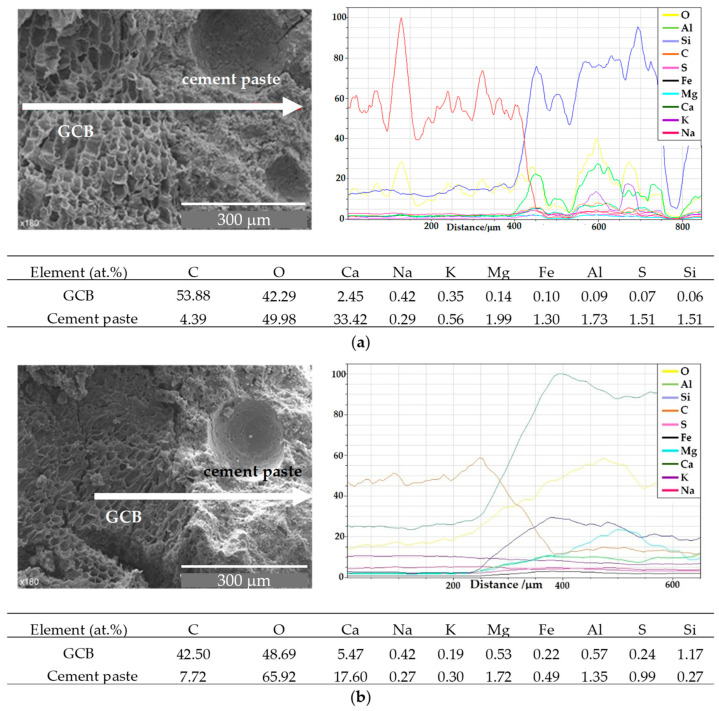
SEM images and EDX analysis of contact area between GCB and cement paste in C3 mix sample (**a**) at 7 days and (**b**) at 28 days.

The variation in the atomic composition along the contact area between the GCB and the cement paste, according to the analysis performed by EDX, is shown in Figure 10a for C3 mix at 7 days and in Figure 10b at 28 days. Although other elements were detected during EDX (e.g., Al, S, Ge, Mg, K, Na) mainly C, Ca and Si best differentiate the microstructural variations at the contact area.

Figure 10a shows a progressive increase in the relative atomic percentage of Ca and Si in a 50 μm transition zone from the GCB aggregate to the cement paste, and a decrease in the percentage of carbon, the main element composing the GCB. A certain proportion of Ca is observed also on the surface of the GCB, since calcium hydroxide crystals, from the cement, are observed inside the cells of the GCB. From this gradual transition we can conclude that there is a good integration of GCB in the cement due to the interaction of some acidic GCB components, such as polyphenols, with the alkaline Ca(OH)_2_, as well as the hydrogen bonds and dispersion forces between the GCB and cement components.

EDX analyses along a 125 µm cement–GCB–cement transition line after 28 days (Figure 10b) show the progressive decrease in the relative percentage of C and the progressive increase in the relative percentage of Ca and Si. This larger scale length of the transition zone identifies an increasing bond strength between the GCB and the cement paste at 28 days in comparison with the bond at 7 days. This agrees with the increasing trend of the mechanical property values in C3 from 7 to 28 days, although these properties showed lower values than in RC [34].

### 3.2. Effects of GCB on Resistance to Extreme Conditions in Non-Structural Concrete

#### 3.2.1. Frost Resistance

Concrete exposed to temperature cycles, in places where water freezes into ice and ice melts into water, is deteriorated due to freezing and thawing, and under these conditions concrete resistance is an issue of great relevance. The cracking and scaling of the concrete surface are the two main degradation processes produced. The resistance of concrete against freeze–thaw cycles is linked to the proportion of harmful pores [50,51] and the pore spacing of the material. In that sense, the greater porosity and cellular structure of the GCB used in this work could benefit the concrete’s durability by the generation of chambers to cushion the pressure increase caused by freezing water. However, a negative effect could be produced if the material favours the increase in internal stresses caused by water accumulation.

**Figure 11:**
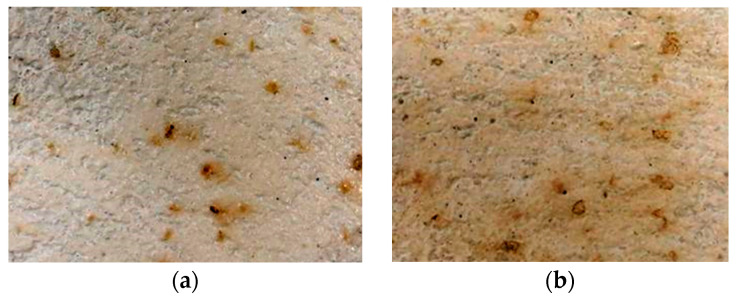
Concrete with GCB: (**a**) surface before freeze–thaw cycles; (**b**) surface after freeze–thaw cycles.

Regarding the visual aspect of the specimens, the concrete surfaces after the freeze–thaw cycles did not show cracks and scaling, similarly to concrete made with corn stalks, as presented in Section 1. Instead, an increase in dark brown spots was observed on the surface of the concrete with the GCB specimens (Figure 11a,b). This fact was more significant on non-moulded surfaces. Water diffusing through the pores of concrete dissolves the Ca(OH)_2_ in the cement paste, creating a strongly alkaline solution. When this solution reaches the GCB, it promotes the degradation of components such as suberin, lignin, cellulose or hemicellulose, resulting in a solution of extractable compounds [23]. These compounds diffuse through the concrete surface surrounding each cork with bark granule, causing stains to appear. Surface yellowing has also been observed in hemp aggregate concretes [52]; however, such spots have not been observed in concrete with non-bio-aggregate like rubber, glass or electric arc furnace slag after freeze–thaw cycles [7,8].

**Figure 12:**
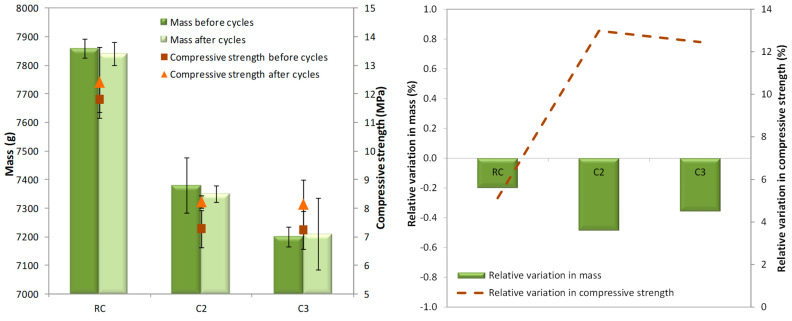
Mass and compressive strength before and after 48 freeze–thaw cycles.

Figure 12 and Table 6 show the masses and the compressive strengths at the beginning and after 48 cycles, and their respective relative variations. Mass values were rounded to the nearest 10 kg/m^3^.

The RC mix presented the highest mass and compressive strength values before and after the freeze–thaw cycles. A decrease in compressive strength from the C2 to C3 mixes was hardly observed as the amount of GCB increased in the specimens. This trend was observed in specimens that had not been subjected to a durability test and in those subjected to freeze–thaw cycles.

The RC, C2 and C3 mixes did not experience loss of compressive strength after 48 freeze–thaw cycles (5.1%, 13.0%, 12%). In a cold environment, the pore water inside the concrete freezes and then melts during the thaw process. Once the freeze–thaw-induced expansion force exceeds the maximum value that the pore can withstand, a crack occurs in the concrete. With the gradual accumulation of such internal damage, the concrete will show gradual freeze–thaw failure [53]. In the concrete with GCB, the expansion force does not exceed the pore’s maximum capacity, allowing the RC, C2, and C3 mixes to withstand the selected test conditions. Moreover, the greater porosity of the concrete with GCB may provide sufficient space for the water to expand without compromising the structure of concrete [54], and the smaller number of smaller pores may help to withstand the selected test conditions because it is known that cracking in concrete during freeze–thaw cycles begins in smaller pores. Concrete with cork shows a similar behaviour to concrete with non-bio-aggregate organic aggregates, such as rubber [55], and better results than concrete made with waste glass. As described in Section 1, concrete with waste glass shows lower frost resistance than the reference concrete. On the other hand, it is observed that the compressive strength values of all concrete mixes after the freeze–thaw cycles were like the compressive strengths obtained at 90 days of age (12.7 MPa, 8.36 MPa, 8.67 MPa). This performance shows again that the GCB does not affect concrete response after 48 freeze–thaw cycles.

Variations in mass before and after the freeze–thaw cycles were not observed. The difference in mass in all the concrete mixes did not exceed 0.5%. That is mainly due to the expansion force not exceeding the maximum value of the pore, meaning that cracks did not occur in the concrete, as discussed above. This behaviour improves upon that of concrete with waste glass [7].

#### 3.2.2. Resistance to Ageing by Thermal Shock

In environments where alternating drying and wetting cycles are significant, such as water level change zones, ocean wave splash zones and tidal zones, during the wetting process a gradient of the different water–soluble components of concrete is released into the interior of the concrete. The drying process increases the concentration of these components in the concrete by water evaporation, with the volume expansion of crystalline products reaching up to four to five times their original size. Thus, a drying–wetting cycle environment is a significant factor to consider in the study of the durability of concrete with GCB since extractable cork components could diffuse into the concrete matrix, affecting the concrete’s material properties [56].

**Figure 13:**
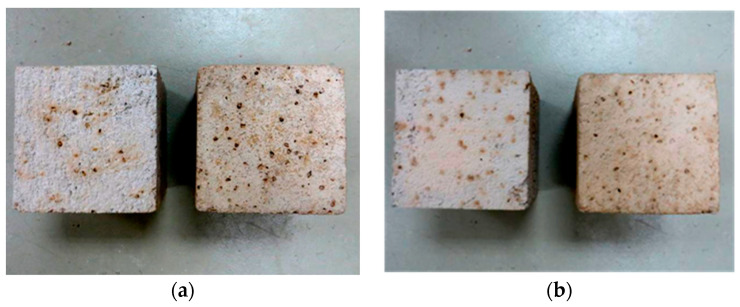
Visual aspect of concrete specimens with GCB before and after wet–dry cycles: (**a**) C2; (**b**) C3.

Figure 13 illustrates the effect of natural aggregate replacement by GCB in the resistance of concrete to 20 wet–dry cycles. After these cycles, the concrete mix surfaces did not show cracks or scaling. However, the concrete specimens with GCB showed a colour change and slight damage in the form of deterioration. Similarly to the frost resistance tests, the colour change in the specimens is attributed to the hydrolysis and dissolution processes of the GCB components by the alkaline aqueous medium generated within the concrete matrix due to the cement hydration diffusion products, primarily Ca(OH)_2_ by water [57]. The concretes in this study have a relatively high porosity that could accentuate the diffusion of the water to reach GCB, as well as the diffusion of the dissolved components.

After the wet–dry cycles, the GCB seems to take on a brownish colour that can bring a feeling of warmth to the concrete and can make the concrete more easily integrated into certain natural environments. It can be considered a positive aspect from the aesthetic point of view. This behaviour has also been observed by González-Ortega et al. [8] in concrete with electric arc furnace slag after wet–dry cycles, which increased the number of stain points. As described in Section 1, water mobilises the products from the corroded iron nodules present in the electric arc furnace slag, transporting and depositing them on the surface of concrete specimens.

**Figure 14:**
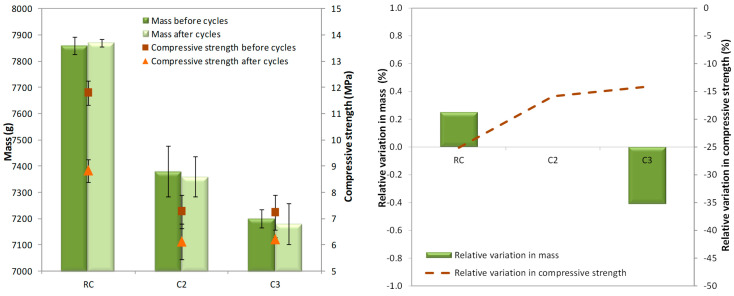
Mass and compressive strength before and after 20 wet–dry cycles.

The mass and the compressive strength of specimens after 20 wet–dry cycles compared with that of specimens that have not been submitted to this durability testing and their relative variations are presented in Table 7 and depicted in Figure 14.

As expected, the RC mix showed the highest mass and compressive strength after the wet–dry cycles. In addition, a decrease in compressive strength from the C2 to C3 mixes was not observed. All the concrete mixes did not lose mass after ageing by thermal shock, but their compressive strengths decreased. In relation to mass, this behaviour can be associated with the relatively stronger interfacial bonding between the Portland-limestone cement (CEM II/A-L 42.25R) used as a binder and GCB, as was shown in the microstructure analysis. Ahmad et al. [31] showed a loss in mass of corn stalk concrete made with magnesium phosphate cement; however, Barbhuiya et al. [58] explains that a good grade and suitable hydraulic binder for hemp concrete increases resistance against wetting and drying cycles. Otherwise, microcracks can occur due to the rapid evaporation of water and expansion at high temperatures of 105 °C, which may affect the strength after wet–dry cycles, but not the mass, as they are not sufficiently aggressive to deteriorate the material. It is important to highlight that compressive strength variation was lower in the concrete with GCB than in the RC mix, which makes it possible to consider applying concrete with GCB in the exterior parts of construction engineering. This behaviour may be attributed to the lower modulus of elasticity of the C2 and C3 mixes that facilitates the absorption of the energy produced by exposure changes and, therefore, there can be more space to reduce deformations or to mitigate cement composite degradation due to wet–dry exposure. On the other hand, extractables from GCB (tannins, waxes, etc.) can positively affect the resistance to wet–dry cycles of concrete with GCB. Similar chemical compounds have been used to seal the pore structure [58].

**Figure 15:**
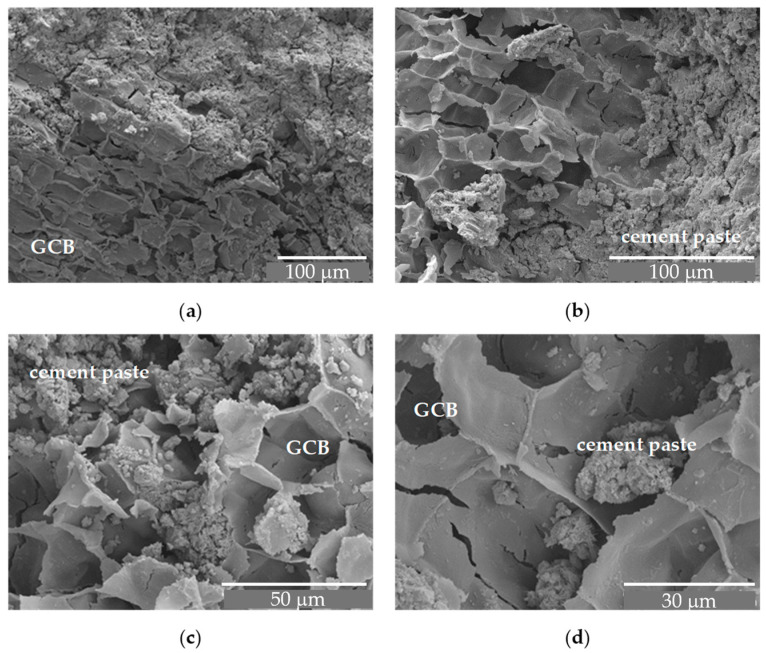
SEM image of C3 sample after wet–dry cycles: (**a**) ×300 magnifications; (**b**) ×500 magnifications; (**c**) ×1000 magnifications; (**d**) ×1500 magnifications.

To better understand the effect of wet–dry cycles on concrete with GCB, its microstructure has been analysed by SEM. Figure 15 shows the microstructure of C3 specimens after 20 wet–dry cycles at different magnifications. A cementitious matrix with an amorphous, dense structure, but with many pores and some cracks that can be attributed to the procedure of obtaining the sample, can be seen in Figure 15a. No needle-shaped crystals (calcium AFt phases) or Ca(OH)_2_ crystals, which do not contribute to a strength decrease, are perceived in these micrographs. Although the GCB maintains its cellular structure, it appears somewhat distorted, with a broken and cracked cell wall in some areas. This deterioration is not observed when the concrete sample has not been subjected to durability tests, as is shown in Section 3.1. This, as mentioned above, may be due to the reaction of the alkaline aqueous solution with the GCB cell wall chemical components. Figure 15b–d correspond to micrographs of the same sample, but they have been taken in different concrete matrix–GCB contact areas. They also show high porosity and coarse pores. The cell wall of the GCB, although it retains its structure, appears cracked and broken.

**Figure 16:**
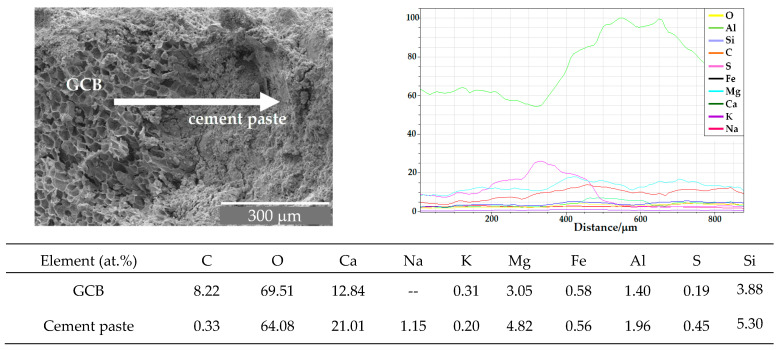
SEM image and EDX analysis of contact area between GCB and cement paste in C3 mix sample after wet–dry cycles.

For cementitious materials, the interfacial transition zone (ITZ) is often considered to be critical to the overall material performance [59]. Figure 16 shows the variation in atomic composition along a line of 875 µm from the aggregate surface of a C3 specimen, according to the microanalyses carried out using EDX. As expected, an increase in the relative percentage of Ca and a decrease in the relative percentage of C, the main GCB element, is observed along a line of around 175 µm from cork granule to cement paste. The relative percentage of oxygen also increases. However, the relative proportion of the rest of the elements remains nearly constant. The ability of water in hydration cement components to dissolve, to extract, to hydrolyse and to diffuse components of GCB explains the presence of carbon on the concrete surface and the stains observed around the cork granules. The length of the line where an atomic composition variation is observed to be about 50 µm wider than that of the samples evaluated before the wet–dry cycles, as was explained in Section 3.1. This difference is owed to the water solution and diffusion of both cork and cement components through both sides of the granule border, favouring the integration of GCB into the cement paste. This matches the lower variation in mechanical strength shown by C2 and C3 after the wet–dry cycles compared to that of RC.

The relative atomic composition of the surface of the GCB and the cement paste near the transition region between the cement paste and the GCB after the wet–dry cycles is shown in Figure 16.

After wet–dry cycles, the relative atomic composition of the GCB surface in the transition region differs from that in specimens that have not been subjected to wet–dry cycles. The different immersions in water favour the diffusion of cement hydration products within the GCB cells and the reprecipitation of these hydration products, likely calcium hydroxide, favouring GCB cell wall mineralization, a better integration of the two materials and a stronger bond between them. This can be observed in the analysis of the relative atomic composition of the cork granule surface. The cork’s main organic component, C, has been practically masked, and its relative atomic percentage has decreased considerably, from 42% to around 8% after the wet–dry cycles. However, the amount of Ca, Mg, Al and Si has increased. A similar effect has been observed by Mohr et al. [57] in kraft pulp fibre–cement composites.

#### 3.2.3. Exposure to High Temperatures

The properties of concrete under high-temperature conditions are crucial for structural safety. While concrete is generally considered non-combustible and fire-resistant, excessive heat and fire can significantly alter its mechanical properties [60]. Therefore, researching the performance and properties of concrete with GCB when exposed to high temperatures is of great interest, particularly due to the potential for spontaneous combustion caused by the presence of GCB in its composition.

**Figure 17:**
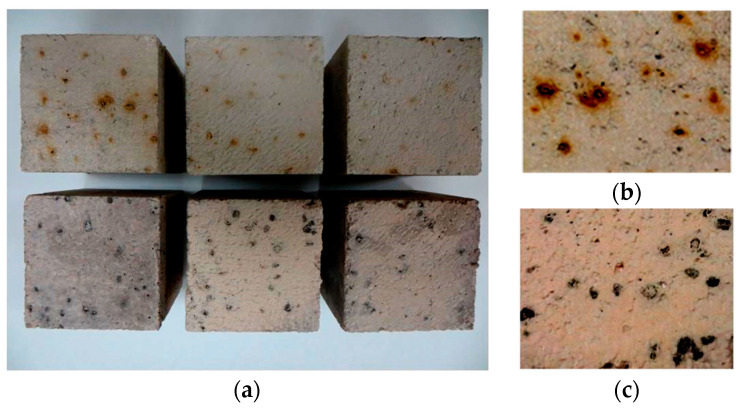
Concrete with GCB: (**a**) specimens before and after exposure to high temperature; (**b**) surface before exposure to high temperature; (**c**) surface after exposure to high temperature.

When the concrete specimens RC, C2 and C3 were exposed to high temperatures, they did not show cracks and scaling. Spontaneous combustion of the concrete with GCB was also not observed. However, a change of colour to light pink was observed in the RC, C2 and C3 specimens after exposure to 600 °C conditions. It is known that when concrete is subjected to high temperatures it undergoes colour changes. Thus, from 300 °C it goes from grey to pink [61,62]. A further change to the grey colour was observed in C2 and C3 at 600 °C, probably due to GCB carbonization (Figure 17a). Furthermore, the disappearing of the stains formed around the GCB is observed in Figure 17b,c. Regarding cork, its organic nature makes it a material strongly sensitive to temperature. Previously, Pereira [63], in a thermogravimetric analysis, observed that cork loses 15% of its mass at 200 °C. This percentage increases rapidly at higher temperatures (27% at 250 °C, 49% at 300 °C, 62% at 350 °C) until ashing at 450 °C. The polysaccharides are its most heat-sensitive components: at 200 °C, hemicellulose disappears and cellulose is degraded to a considerable extent. Suberin is more resistant, and degradation starts at approx. 250 °C; 300 °C treated samples only contain 7% suberin. With reference to concrete, it is observed that its characteristic surface gloss disappeared due to surface exudation alongside its change to a pinkish colour after high-temperature exposure. As presented in Section 1, colour changes in concretes with oil palm shell and coconut shell aggregates were also observed upon exposure to elevated temperatures [28,29,30].

**Figure 18:**
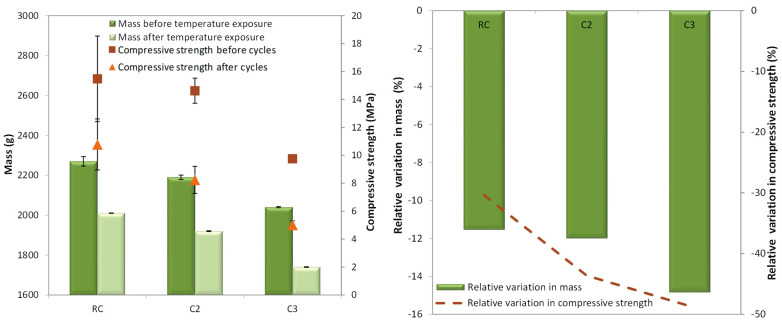
Mass and compressive strength before and after exposure to high temperatures.

Table 8 and Figure 18 show the mass, and the compressive strength of concrete mixes subjected to elevated temperature, in addition to the relative variations in these measurements. The RC mix presented the highest mass and compressive strength values before and after exposure to high temperatures. The decrease in mass and compressive strength of the mixes with GCB show similar values regardless of the amount of GCB.

All concrete mixes subjected to high temperatures showed a decrease in mass. This decrease was about 11% for the CR mix and between 12% and 15% for the concrete mixes with GCB. The C3 mix showed a slightly higher mass variation, possibly because of having a greater quantity of GCB in its composition and, therefore, a greater degradation of its granules. The mass loss in concrete at high temperatures has been assigned to the decomposition of calcareous aggregates, liberation of carbon dioxide (CO_2_) and sloughing of the concrete surface [64,65]. GCB, having an organic nature, starts to lose mass at 200 °C, as was mentioned above, and the process continues until its complete degradation.

Mixes C2 and C3 presented a greater decrease in compressive strength than the RC concrete after high-temperature exposure. This decrease was about 30% for the RC mix and between 43% and 49% for the concrete mixes with GCB. At low temperatures, the cement paste expands when heated, but at 300 °C, shrinkage occurs due to the loss of water. Conventional aggregates continue to expand with temperature and, therefore, this leads to a loss of strength [45]. Regarding concrete mixes with GCB, the loss of compressive strength can be attributed to GCB carbonization. The residual compressive strength of the RC mix (around 70%) was higher than that of the concrete mixes with GCB (around 60%). A similar behaviour has been described for lightweight concrete made with limestone aggregate [62] or PET waste concrete [66] after they were exposed to high temperatures. The more porous structure of PET waste concrete leads to an imbalanced thermal gradient, causing the formation of cracks and/or the thermal degradation of the PET. However, Mohammed et al. [9] described that concrete made with EPS exhibited better compressive strength than the reference concrete after burning due to the EPS creating a medium where bonding is still available between the composing materials even after burning.

On the other hand, the compressive strength loss for these concrete mixes prepared with GCB is not as high as the compressive strength loss of concrete mixes with oil palm or coconut shell aggregates prepared by Mo et al. [27] and Gunasekaran et al. [29] and exposed to 400 °C conditions.

The mass losses and strength decreases in C2 and C3 with respect to the RC mix when subjected to high temperatures are between 4 and 14% and 16 and 50%, respectively. They were slightly higher in the case of mass loss and greater in the case of compressive strength decrease compared to the values obtained for the concrete mixes that were not exposed to high temperatures (3–11% and 5–39%).

**Figure 19:**
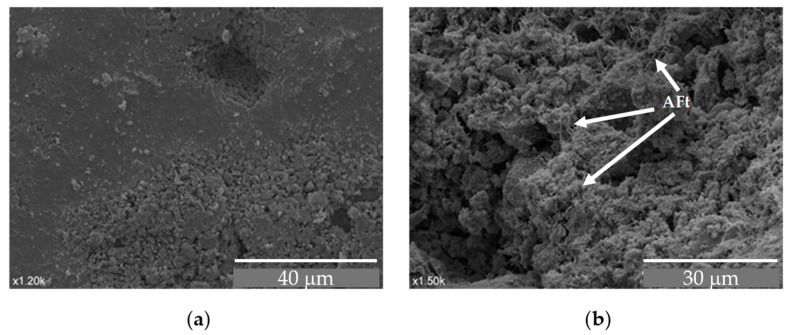
SEM images of C3 mix sample after exposure to high temperatures: (**a**) more dense and compact structure × 1200 magnifications; (**b**) porous structure × 1500 magnifications.

Figure 19 shows the topography of the C3 mix in two visually different areas, one denser and more compact formed next to the mould wall (surface in contact with the mould) and the other one having numerous pores. Both show a deteriorated structure.

Figure 19a shows that the cement has an amorphous structure, which may be due to a mixture of CSH, calcium hydroxide and AFm phases. However, Figure 19b shows a structure that is covered with acicular structures typical of calcium AFt phases which appear in less-advanced cement hydration processes. In this case, the decomposition processes of calcium hydroxide at 450–550 °C and the generation of water vapour in the internal matrix of the concrete due to high temperatures produced a hydration process by a successive lowering of temperatures, leading to the formation of calcium AFt phases again [67].

**Figure 20:**
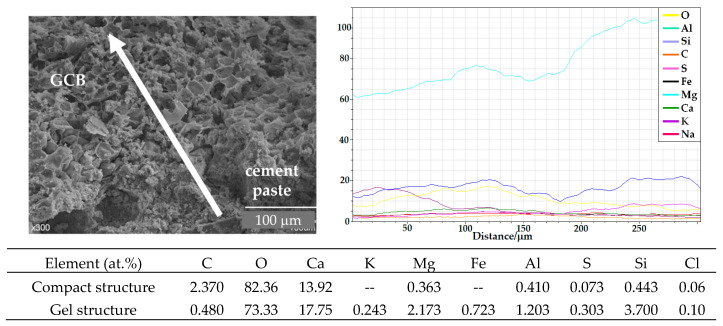
SEM image and EDX analysis of contact area between GCB and cement paste in C3 mix sample after exposure to high temperatures.

Figure 20 shows the variation in the atomic composition along a line of 300 µm in the transition region between cement paste and GCB of the C3 mix, according to the microanalyses carried out using EDX. Although the image shows the presence of both materials, cork and concrete, in the transition region, the surface analysis shows a homogeneous composition along the line, although the relative amount of Si decreases along the line from concrete matrix to GCB and the relative amount of Ca increases. High temperatures, as well as rehydration processes due to the presence of water vapour during cooling, favour the diffusion of the different components of the cement paste towards the GCB. Then, after high temperatures, the contact zone between GCB and concrete shows a very good integration of the two materials. According to this, the mechanical strength decrease after exposure to high temperatures should be less than after wet–dry cycles, but it is not, probably due to the loss of cork mass.

## 4. Conclusions

The evidence provided in this study suggests that GCB can be used as an aggregate to produce concrete for use in non-structural precast elements. In fact, the durability assessed for the non-structural concrete with the GCB used in this work exhibits good quality in terms of frost resistance and ageing by thermal shock. Additionally, it exhibits acceptable quality when exposed to high temperatures.

1. The microstructure shows a good integration of the GCB in the cement matrix, forming dense and compact structures. In addition, the different phases of hydrated cement can be seen around the GCB.
2. The resistance of the concrete mixes with GCB against 20 freeze–thaw cycles is like that of the RC mix. No signs of cracks or scaling were observed in the concrete mixes with GCB and the RC mix, although an increase in dark brown spots was observed on the surface of the GCB specimens.
3. The concrete mixes with GCB after wet–dry cycles showed a colour change and slight deterioration. The concrete mixes with GCB presented a similar variation in mass and a lower reduction of compression strength (around 15%) to that of the RC mix (around 25%) after 20 wet–dry cycles. The microstructure shows that cell wall of the GCB, although it retains its structure, appears cracked and broken.
4. In terms of resistance towards elevated temperature, the surfaces of the concrete mixes with GCB show a colour variation to light pink followed by a second change to black. Spontaneous combustion of the concrete with GCB was not observed. Variations in mass and compressive strength of the concrete mixes with GCB with elevated temperature are greater than that of the RC. The microstructure shows a deteriorated structure with acicular crystals typical of calcium AFt phases. These appear in poorly advanced cement hydration processes.

This study shows the potential of GCB for use as an aggregate in concrete for non-structural precast products. However, further research is required to evaluate its long-term performance (sulphate resistance, acid resistance, etc.) to optimise its use due to the variety of non-structural precast products. For practical applications, an industrial scale study is essential.

## Figures and Tables

**Table 1:** Granulometry of GCB and limestone raw aggregates.

Size Fraction	GCB (%)	0–4 mm (%)	2–5 mm (%)	4–8 mm (%)
10 mm	-	100	100	100
8 mm	-	99.7	100	99.8
6.3 mm	100	99.4	99.9	72.3
5 mm	99	98.6	97.8	23.9
4 mm	64.3	96.9	72.7	5.8
3.2 mm	3.9	-	-	-
2 mm	-	73.1	6.7	2.2
1 mm	-	50.1	2.8	2.1
0.5 mm	-	32.2	2.6	2.0
0.25 mm	-	21.4	2.4	2.0
0.125 mm	-	13.0	2.3	1.9
0.063 mm	-	6.3	2.1	1.8

**Table 2:** Physical properties of GCB and limestone raw aggregates.

Property	GCB	Aggregates		
		0–4 mm	2–5 mm	4–8 mm
Apparent particle density (kg/m^3^)	573.6	2823	2782	2812
Oven-dried particle density (kg/m^3^)	388.8	2769	2733	2728
Saturated and surface-dried particle density (kg/m^3^)	712.2	2788	2750	2758
Water absortion after 24 h (%)	83.2	0.69	0.64	1.09
Porosity (%)	29.0	0.04	0.03	0.08

**Table 3:** Composition of concrete mixes (kg/m^3^).

Mixes	Cement	Fine Aggregate	Coarse Aggregate	GCB	Coarse Aggregate	Water
		0–4 mm	2–5 mm	3.2–5 mm	4–8 mm	
RC	182	1365	331	---	331	218
C2	182	1361	264	15	330	218
C3	181	1359	231	23	329	217

**Table 4:** Porous structure of RC, C2 and C3 [35].

Mixes	Porosity (%)				Middle Pore Radius (µm)
	Total	Gel Pore	Capillary Pore	Capillary Pore	
		Capillary Pore		Entrained Air	
		0.01–0.1 (µm)	0.1–10 (µm)	10–100 (µm)	
RC	16.7	8.1	7.6	1.1	0.036
C2	19.3	7.5	10.4	1.8	0.052
C3	21.1	5.9	13.4	2.7	0.064

**Table 5:** Mechanical properties of RC, C2 and C3 [35].

Mixes	Flexural Strength (MPa)			Compressive Strength (MPa)			Elastic Modulus (MPa)
	7 Days	28 Days	90 Days	7 Days	28 Days	90 Days	
RC	2.6 + 0.3	3.2 + 0.2	3.3 + 0.4	8.7 + 0.8	11.8 + 0.5	12.7 + 0.9	14,500
C2	2.3 + 0.1	3.0 + 0.3	3.2 + 0.2	5.5 + 0.2	7.3 + 0.6	8.4 + 0.1	12,000
C3	1.6 + 2.1	2.1 + 0.1	2.3 + 0.1	5.6 + 0.5	7.2 + 0.7	8.7 + 0.4	10,500

**Table 6:** Mass and compressive strength before and after 48 freeze–thaw cycles.

Mixes	Mass (g)		Relative Variation in Mass (%)	Compressive Strengh (MPa)		Relative Variation in Compressive Strength (%)
	Before Freeze–Thaw Cycles	After Freeze–Thaw Cycles		Before Freeze–Thaw Cycles	After Freeze–Thaw Cycles	
RC	7860 ± 33	7840 ± 41	−0.2	11.8 ± 0.5	12.4 ± 1.2	5.1
C2	7380 ± 97	7350 ± 28	−0.5	7.3 ± 0.6	8.2 ± 0.2	13.0
C3	7200 ± 34	7210 ± 126	−0.4	7.2 ± 0.7	8.1 ± 0.9	12.5

**Table 7:** Mass and compressive strength before and after 20 wet–dry cycles.

Mixes	Mass (g)		Relative Variation in Mass (%)	Compressive Strengh (MPa)		Relative Variation in Compressive Strength (%)
	Before Wet–Dry Cycles	After Wet–Dry Cycles		Before Wet–Dry Cycles	After Wet–Dry Cycles	
RC	7860 ± 33	7870 ± 14	−0.1	11.8 ± 0.5	8.8 ± 0.4	−25.1
C2	7380 ± 97	7360 ± 77	0.3	7.7 ± 0.6	6.1 ± 0.7	−15.9
C3	7200 ± 34	7180 ± 79	0.3	7.2 ± 0.7	6.2 ± 0.1	−14.2

**Table 8:** Mass and compressive strength before and after exposure to high temperature.

Mixes	Mass (g)		Relative Variation in Mass (%)	Compressive Strength (MPa)		Relative Variation in Compressive Strength (%)
	Before Exposure to High Temperature	After Exposure to High Temperature		Before Exposure to High Temperature	After Exposure to High Temperature	
RC	2270 ± 24	2010 ± 1	−11.6	15.5 ± 3.1	10.8 ± 1.8	−30.4
C2	2190 ± 11	1920 ± 2	−12.0	14.6 ± 0.9	8.24 ± 1.0	−43.6
C3	2040 ± 3	1740 ± 2	−14.9	9.8 ± 0.1	5.0 ± 0.3	−48.6

## Data Availability

The original contributions presented in this study are included in the article. Further inquiries can be directed to the corresponding author.
